# Rapid Discrimination of *Pseudomonas aeruginosa* ST175 Isolates Involved in a Nosocomial Outbreak Using MALDI-TOF Mass Spectrometry and FTIR Spectroscopy Coupled with Machine Learning

**DOI:** 10.1155/2023/8649429

**Published:** 2023-09-07

**Authors:** Ana Candela, Manuel J. Arroyo, María Sánchez-Cueto, Mercedes Marín, Emilia Cercenado, Gema Méndez, Patricia Muñoz, Luis Mancera, David Rodríguez-Temporal, Belén Rodríguez-Sánchez

**Affiliations:** ^1^Clinical Microbiology and Infectious Diseases Department, Hospital General Universitario Gregorio Marañón, Madrid, Spain; ^2^Instituto de Investigación Sanitaria Gregorio Marañón, Madrid, Spain; ^3^Clover Bioanalytical Software, Av. del Conocimiento, 41, Granada 18016, Spain; ^4^CIBER de Enfermedades Respiratorias (CIBERES CB06/06/0058), Madrid, Spain; ^5^Medicine Department, Faculty of Medicine, Universidad Complutense de Madrid, Madrid, Spain

## Abstract

The goal of this study was to evaluate matrix-assisted laser desorption ionization–iime of flight mass spectrometry (MALDI-TOF MS) and Fourier-transform infrared spectroscopy (FTIR-S) as diagnostic alternatives to DNA-based methods for the detection of *Pseudomonas aeruginosa* sequence type (ST) 175 isolates involved in a hospital outbreak. For this purpose, 27 *P. aeruginosa* isolates from an outbreak detected in the Hematology department of our hospital were analyzed by the above-mentioned methodologies. Previously, these isolates had been characterized by pulse-field gel electrophoresis (PFGE) and whole-genome sequencing (WGS). Besides, eight *P. aeruginosa* isolates were analyzed as unrelated controls. MALDI-TOF MS spectra were acquired by transferring several colonies onto the MALDI target and covering them with 1 *µ*l of formic acid 100% and 1 *µ*l of *α*-ciano-3,4-hidroxicinamic acid matrix. For the analysis with FTIR-S, colonies were resuspended in 70% ethanol and sterile water according to the manufacturer's instructions. Spectra from both methodologies were analyzed using Clover Biosoft Software, which allowed data modeling using different algorithms and validation of the classifying models. Three outbreak-specific biomarkers were found at 5,169, 6,915, and 7,236 *m/z* in MALDI-TOF MS spectra. Classification models based on these three biomarkers showed the same discrimination power displayed by PFGE. Besides, *K*-nearest neighbor algorithm allowed the discrimination of the same clusters provided by WGS and the validation of this model achieved 97.0% correct classification. On the other hand, FTIR-S showed a discrimination power similar to PFGE and reached correct discrimination of the different STs analyzed. In conclusion, the combination of both technologies evaluated, paired with machine learning tools, may represent a powerful tool for real-time monitoring of high-risk clones and isolates involved in nosocomial outbreaks.

## 1. Introduction

Healthcare-associated infections are becoming one of the major health concerns of the 21st century. This term groups together infections developed during and/or resulting from a hospital or nursing home stay that were not detected at the time of admission [1]. They represent the most frequent adverse event in healthcare settings (6.5% in acute care hospitals in the European Union and 3.2% in hospitalized patients in the United States) [2, 3]. Pathogens that cause nosocomial infections can spread and cause outbreaks among inpatients and staff members, requiring control and treatment measures and ultimately increasing resource costs in hospital settings [4]. The emergence of multidrug resistant microorganisms is another concern that arises in nosocomial outbreaks, as they pose an added complication in relation to the correct choice of antimicrobial treatment [5]. The control of nosocomial infection caused by multiresistant bacteria and outbreak surveillance programs should be implemented in hospitals in order to reduce mortality/morbidity, length of stay, and hospital costs [6].

*Pseudomonas aeruginosa* is one of the most frequent pathogens involved in outbreaks in hospitals and long-term care facilities [7]. This microorganism is an environmental, gram-negative, nonfermenting bacterium that can easily become a multidrug-resistant (MDR) and extensively drug-resistant pathogen through mutations in chromosomal genes in addition to its intrinsic resistance mechanisms [8]. Its wide range of virulence factors, such as its ability to produce biofilm and also its capacity to persist in moist environments, such as sinks and shower plates in hospitals, coupled with its clonality and fast spread of high-risk clones, make it an ideal candidate for being the cause of nosocomial outbreaks [9, 10].

The reference method for outbreak characterization remains pulse-field gel electrophoresis (PFGE) but it may be insufficient for clone discrimination in some cases [11]. Multilocus sequence typing (MLST) provides complementary information to PFGE but it is still laborious and time consuming [12]. Although the implementation of novel approaches such as whole-genome sequencing (WGS) would improve the follow-up of clinical outbreaks by increasing the quantity and quality of the information obtained, it requires expensive and sophisticated equipment and highly skilled personnel, making it unaffordable in most clinical laboratories nowadays.

Matrix-assisted laser desorption ionization–time of flight mass spectrometry (MALDI-TOF MS) is currently implemented in most clinical microbiology laboratories for bacterial species identification [13]. This technology has also been evaluated for purposes beyond identification, such as antimicrobial resistance detection or bacterial typing [14–16]. Therefore, MALDI-TOF MS could be a rapid and available alternative for outbreak characterization [17, 18]. In addition, Fourier-transform infrared spectroscopy (FTIR-S) has emerged as a promising new tool for bacterial typing [19]. This methodology has been recently applied to *Streptococcus pneumoniae* typing [20].

In this study, we evaluated MALDI-TOF MS and FTIR-S coupled with machine learning classification methods for the rapid detection and follow-up of a nosocomial outbreak caused by a *P. aeruginosa* high-risk clone with the same level of accuracy provided by advanced molecular methods.

## 2. Materials and Methods

### 2.1. Outbreak Description and Bacterial Isolates

Between October 2013 and December 2014, a nosocomial outbreak caused by *P. aeruginosa* showing an MDR phenotype was detected in the Hematology ward of a Spanish tertiary hospital (Hospital General Universitario Gregorio Marañon (HGUGM), Madrid, Spain; 1,350 beds) [21]. Patients hospitalized at this ward showed an increased incidence of *P. aeruginosa* bacteremia, while this was not reflected at the overall hospital level. During this 15 month period, at least one isolate of *P. aeruginosa* showing the same MDR phenotype was detected in 14 patients, while this microorganism was not found in the same ward during the previous 8 months. The isolates were resistant to carbapenems (imipenem and meropenem), antipseudomonal fluoroquinolones (ciprofloxacin and levofloxacin), and aminoglycosides (gentamycin and tobramycin), fulfilling the criteria established by Magiorakos et al. [22] for MDR microorganisms.

As a first approach, PFGE was performed on 23 *P. aeruginosa* isolates: 12 available isolates out of the 14 outbreak-suspected strains from 2013 to 2014, 2 of them sourcing from the same patient; 5 controls strains related to the Hematology ward and 6 unrelated strains from the same time period. Moreover, a reference *P. aeruginosa* strain (ATCC 27853) was also included in the analysis in duplicate [21]. The methodology applied for PFGE has been described before [23]. Briefly, the strains were digested with SpeI overnight at 37°C and the DNA fragments were separated by electrophoresis in a Chef DRII instrument (Bio-Rad Laboratories, Inc., Hercules, CA) using a 1% agarose gel in 0.5X Tris-borate-EDTA buffer. The fingerprints obtained were analyzed with Bionumerics Software 4.0 (bioMérieux, Marcy-l'Étoile, France). Strains were considered to be identical when 99.9% similarity was achieved.

**Figure 1:**
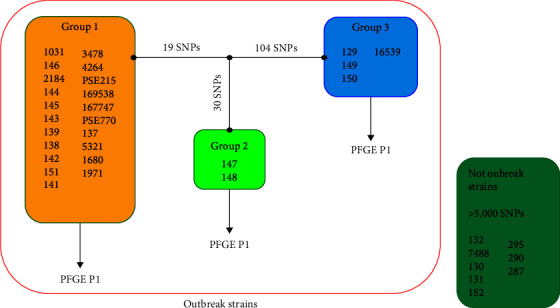
Classification of *Pseudomonas aeruginosa* strains as “outbreak” by pulse-field gel electrophoresis (PFGE) and “not outbreak” (in dark green). The three different groups obtained by whole-genome sequencing (WGS) are shown: Group 1, where the outbreak isolates are clustered; Group 2, containing two isolates separated by 30 single nucleotide polymorphisms (SNPs) from Group 1; and Group 3, where 4 isolates separated by 104 SNPs from Group 1 clustered. According to WGS, Groups 2 and 3 are not part of the outbreak.

The strains that grouped in the outbreak-pulsotype (P1) were further characterized by WGS (Table S1). This methodology differentiated three clusters within pulsotype P1: Group 1, which contained the *P. aeruginosa* strains correlated to the outbreak, and Groups 2 and 3, where isolates detected during the same period as the outbreak strains but with enough single nucleotide polymorphisms (SNPs) to be considered as nonoutbreak strains clustered (Figure 1) [21].

With the outbreak-specific SNPs detected by WGS analysis, a multiplex allele-specific oligonucleotide polymerase chain reaction (ASO-PCR) was designed for the rapid differentiation of the outbreak-related *P. aeruginosa* isolates (Group 1). The ASO-PCR was tested with a collection of isolates (*n* = 32) from a broader period of time (2010–2018), allowing for the detection of new outbreak-related strains which were not considered part of the outbreak initially (Figure 1) [21].

A total of *n* = 67 available *P. aeruginosa* strains from this outbreak were included in this study (Table S1): 35 *P. aeruginosa* isolates analyzed by WGS (27 outbreak strains–all of them ST175–, and 8 control strains; Figure 1) and 32 isolates (16 outbreak and 16 nonoutbreak strains) analyzed by ASO-PCR. All strains sourced from inpatients from HGUGM and were kept frozen at −80°C until further analysis. Isolates were cultured on 5% Columbia sheep blood agar plates at 37°C in aerobic conditions, metabolically activated after three passages and analyzed after a 24 hr overnight incubation period.

### 2.2. MALDI-TOF MS Identification

A small amount of biomass from each isolate was spotted onto the MALDI target plate, covered with 1 *μ*l of formic acid 100% for on-plate protein extraction and allowed to dry. Then, 1 *μ*l of organic matrix (*α*-ciano-3,4-hidroxicinamic acid) was added and allowed to dry again before MALDI-TOF MS analysis. Strains were identified with an MBT Smart MALDI Biotyper (Bruker Daltonics, Bremen, Germany) using the updated database containing 9,957 reference spectra profiles.

### 2.3. MALDI-TOF MS Spectra Processing and Data Modeling

Spectra were acquired using default settings and visualized with FlexAnalysis Software (Bruker Daltonics), where outliers and zero lines were removed. MALDI-TOF MS spectra were exported to and further processed with Clover MS Data Analysis Software (Clover Biosoft, Granada, Spain) as follows: (a) variance stabilization, (b) smoothing by Savitzky–Golay filter (window length: 11, polynomial order: 3), (c) baseline subtraction using top-hat filter (0.02), and (d) TIC (total ion current)-normalized (Figure S1). Replicated peaks were aligned in the 2,000–20,000 Daltons region of the spectra—were most bacterial proteins can be found—and then merged in an average spectrum for each isolate according to the information compiled in a previous study [24].

As a first approach, the mass spectra from the 35 strains characterized by WGS were used as the training set for data modeling. Two peak matrices were built: (a) using the threshold method, which consisted of applying a 0.01 threshold value to average spectra, which selected only the peaks above 1.0% of the maximal intensity (Prominence: 0.01; Distance: 1) within 2,000–20,000 Daltons range and (b) using the biomarker selection method, that searches for specific peaks for each category (“outbreak” or “control” *P. aeruginosa* isolates). Peaks within the 2,000–10,000 Daltons range and with an area under the received operating characteristic curve (AUROC) higher than 0.85 were evaluated and selected for the construction of the biomarker peak matrix (Figure S1). The AUROC values were obtained by matching all samples of both categories with peaks above 0.01 threshold.

Matrices built using both the threshold and biomarker selection methods were used as input data for training machine learning: first, two unsupervised algorithms—principal component analysis (PCA) and hierarchical clustering—were applied to check the clustering between samples and categories and settle their distances in a dendrogram and second, different supervised algorithms—partial least squares discriminant analysis (PLS-DA); linear support vector machine (SVM); random forest (RF) and neighborhood components analysis with *K*-nearest neighbors (NCA-KNN)—were used as classifiers to build predictive models. Supervised algorithms training was carried out by systematically testing different combinations of values for their respective hyperparameters. Each hyperparameter is given a list of possible values, and all combinations are tested for training. The combination which achieved the highest balanced accuracy was chosen. For SVM, we automatically tuned the hyperparameter C, which controls the trade-off between the training error and the classification error on unseen data. Thus, a large hyperparameter C value indicates a highly strict and potentially overfitting model whilst a lower value leads to a more heavily regularized model which will allow more misclassifications in the training set in exchange for a better generalization to unobserved samples. For NCA-KNN, this optimization is performed on the number of neighbors used (*K*). On the other hand, for RF we automatically tested a range of potential values in the following hyperparameters: number of trees in the forest, number of features to consider when looking for the best split, maximum depth of each tree, minimum number of samples required to split an internal node, and the minimum number of samples required to be a leaf node. Details on the values explored can be found in Table S2. Cross-validation was performed for each model by *k*-fold method (*k* = 10) as described previously [16] and leave-one-out (LOO) method (Figure S1). The *k*-fold method divides the dataset into *k*-stratified folds and tests all folds as the validation set using the remaining folds as the training set. With the LOO method, each sample is used as a single set for validation and the rest as the training set. The final configuration of each supervised algorithm was saved as a prediction model to be validated.

For the validation of the predictive models described in the previous paragraph, a total of 32 isolates, 15 “outbreak” and 17 “control,” analyzed by ASO-PCR were included in the validation set. Average spectra obtained from four replicates analyzed twice for each isolate were preprocessed as described in the creation of predictive models and used as input data to be automatically classified by the prediction models.

### 2.4. Reproducibility Assessment

A reproducibility assay was carried out with the 35 *P. aeruginosa* strains characterized by WGS. Each isolate was spotted in quadruplicate (technical replicates) to assess the interspot or the technical reproducibility of the method. Besides, two spectra were acquired per spot to make an average spot spectrum. The biological reproducibility was analyzed by subculturing overnight under the same conditions as indicated above and acquiring protein spectra again in quadruplicates [24, 25]. Data analysis was carried out with Clover MS Data Analysis Software. The pipeline described above was applied to the acquired spectra, which were subsequently aligned to obtain an average spectrum per spot from two spectra per spot (shift medium; linear mass tolerance 200 ppm). The same procedure was applied to obtain a single average spectrum per day from four spots for each isolate. This approach allowed the detection of common peaks present in all protein spectra. The coefficient of variation (% CV) of the peak intensities registered for each common peak after a 0.1 threshold applied was calculated from TIC-normalized spectra.

### 2.5. FTIR-S Spectra Acquisition and Processing

FTIR-S was performed only on the strains which had combined PFGE plus WGS information in the initial study by Acosta et al. [21] (*n* = 20). FTIR-S spectra acquisition was performed using IR Biotyper (Bruker Daltonics) following the manufacturer's instructions. Briefly, a 1 *μ*l loopful of biomass was resuspended in 50 *µ*l of 70% ethanol and homogenized with metal rods (Bruker Daltonics). Then, 50 *µ*l of sterile water were added and 15 *µ*l of the suspension were spotted on a silicon plate. Samples were analyzed in triplicates in three independent experiments along with two standards (Bruker Infrared Test Standard 1 and 2, Bruker Daltonics). FTIR-S spectra were visualized and processed using Clover MS Data Analysis Software. The processing consisted on standard normal variation followed by Savitzky–Golay filter with nine smoothing points, polynomial order = 2 and second derivative using the entire wavenumbers range. Moreover, spectra with valid target quality controls were further analyzed using hierarchical cluster analysis (HCA) with Euclidean distance and ward metric and PCA to evaluate clustering of isolates according to molecular techniques (Figure S1).

## 3. Results

A preliminary version of the results shown in this article have been previously published as a preprint [26].

### 3.1. MALDI-TOF MS Analysis

All strains analyzed in this study (*n* = 67) were identified by MALDI-TOF MS as *P. aeruginosa* with a score ≥2.0, showing, therefore, correct species-level identification according to the manufacturer's criteria [27]. Besides, this identification was the only one provided by MALDI-TOF MS along the top 10 identifications given for each isolate.

#### 3.1.1. Analysis of Outbreak Strains Characterized by WGS

**Figure 2:**
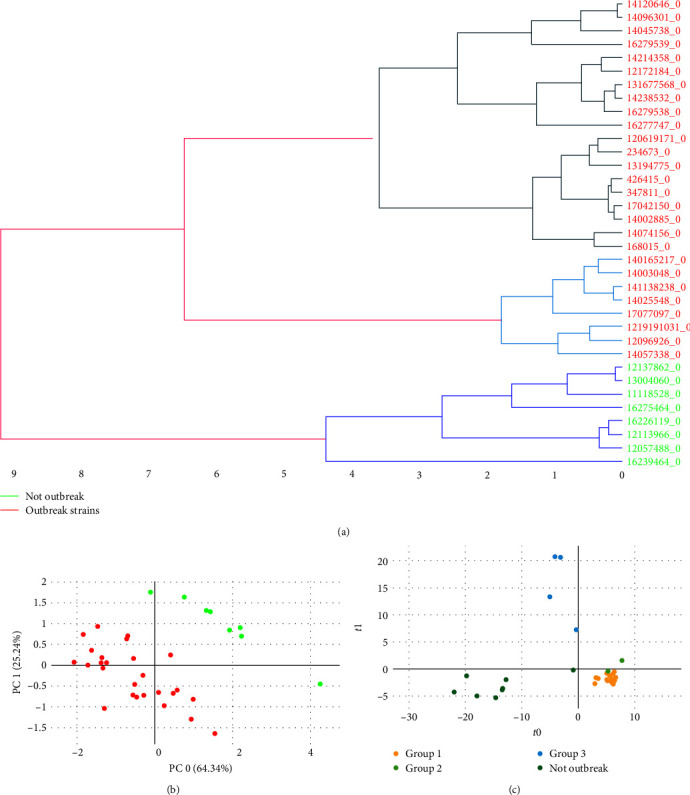
Classification of MALDI-TOF MS spectra between “outbreak” and “not outbreak” isolates. (a) Hierarchical clustering analysis with Euclidean distance and ward metric, (b) principal component analysis, and (c) differentiation of WGS-groups by PLS-DA (partial least squares discriminant analysis) algorithm. MALDI-TOF MS, matrix-assisted laser desorption ionization–time of flight mass spectrometry; WGS, whole-genome sequencing.

Firstly, a peak matrix was constructed using MALDI-TOF MS spectra from the strains previously analyzed by WGS (*n* = 35) by applying the threshold method with a total of 413 peaks as features. The isolates were initially classified according to PFGE results, where the strains were grouped as P1 (outbreak) and other pulsotypes considered as unrelated strains. The cross-validation of this approach (*k* = 10) yielded 97.1% isolates correctly classified using PLS-DA, RF, and NCA-KNN algorithms and 94.3% with SVM (Table S3). Besides, using the biomarker selection method three potential biomarkers were found at 5,169, 6,915, and 7,236 *m/z*. The peak matrix constructed with these three peaks correctly classified all strains (100%) in all prediction models tested (PLS-DA, SVM, RF, and NCA-KNN) by *k*-fold validation (*k* = 10). The implementation of unsupervised algorithms also achieved optimal separation of the two main categories (“outbreak” and “control” strains) displaying two well-defined clusters in the PCA plot and HCA dendrogram (Figure 2).

In a second step, MALDI-TOF MS spectra were further compared according to WGS clustering, where the outbreak strains clustered by PFGE in the pulsotype 1 (P1) were divided into three outbreak groups: Group 1, considered the main outbreak strains, Group 2 and 3 (separated by <125 SNPs from Group 1) and Controls (>5.000 SNPs difference) (Figure 1). Differentiation of what WGS considered the main outbreak (Group 1) from the rest of the strains (“Controls,” “Group 2,” and “Group 3”) was attempted in this step. For this purpose, a peak matrix was created by applying the threshold method and used as input data to PLS-DA, SVM, RF, and NCA-KNN algorithms. They obtained a correct classification of 94.3% by SVM (*C* optimized hyperparameter: 0.01), 85.7% by PLS-DA and NCA-KNN (NCA component reduced to 3, neighbors optimized hyperparameter: 3), and 88.6% by RF (number of estimators optimized hyperparameter: 100) (Table S4; Figure S2). Group 2 strains (*n* = 2) appeared closer to the outbreak strains than Group 3 and control strains (Figure 2(c)), as it is closer to the Group 1 strains in a number of SNPs (50 SNPs).

#### 3.1.2. External Validation of the Initial Model

Lastly, the biomarker classification model—SVM, three biomarker peaks—(*C* optimized hyperparameter: 0.19) developed with the strains characterized by WGS was blindly validated with the isolates characterized by ASO-PCR (*n* = 32: 15 “outbreak” and 17 “control”). The validation of the model yielded 90.0% correct classification of the strains, misclassifying only 3/32 ASO-PCR strains (two outbreak isolates as nonoutbreak, and one nonoutbreak as outbreak strain).

#### 3.1.3. Reproducibility of the Method

The comparison of the 13 common peaks from protein spectra obtained from the different spots in which the same isolate was smeared (interspot variability) showed 11.0% and 14.5% of mean of their CVs values for day 1 and day 2, respectively. Besides, the analysis of the variance yielded a maximum of 20.5% CV for the most common peaks detected within the 7,000–10,000 *m/z* range, although most peaks between 2,000 and 7,000 *m/z* interval showed CV values between 10.0% and 15.0% (Figure S3(a)). The mean and median interday CV values were 9.2% and 5.8%, respectively. CV values varied between 0.02% and 18.0%, a range considered as acceptable by Pang et al. [28] (Figure S3(b)). The only exception here was the 5,740 *m/z* peak, whose interday CV was almost 30.0%.

The reproducibility of the specific biomarkers that allowed the differentiation of the *P. aeruginosa* isolates belonging to the outbreak was further analyzed. In this case, three peaks were identified as potential biomarkers for differentiation between outbreak strains and those not related with the outbreak (peaks 5,169, 6,915, and 7,236 *m/z*). The average intraday CV for these peaks was 13.6% and this value varied between 7.0% and 20.5% (Figure S3(c)). However, the biomarker peak at 6,915 *m/z* showed 32.5% interday CV variability (Figure S3(d)).

### 3.2. FTIR-S Analysis

**Figure 3:**
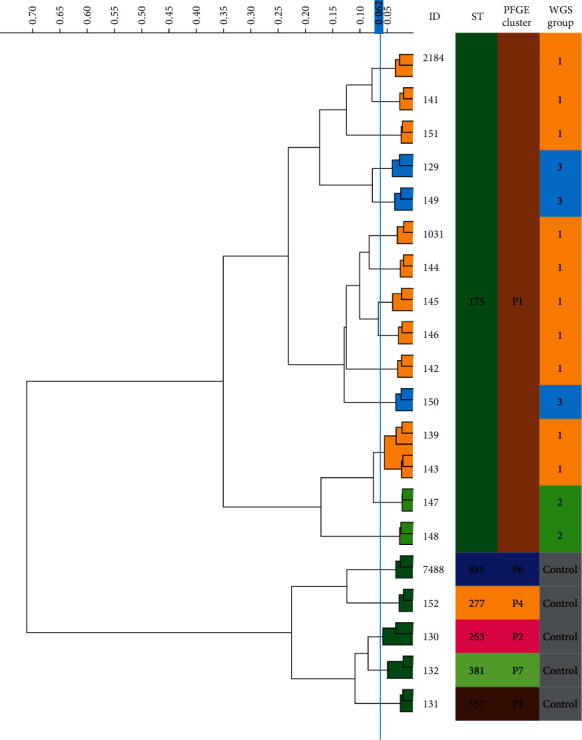
Dendrogram obtained by IR Biotyper for *Pseudomonas aeruginosa* isolates analyzed in the lipids region (1,400–1,500 and 2,800–3,000 cm^−1^). Hierarchical cluster analysis with Euclidean distance and ward metric was performed. ST, sequence type; PFGE, pulsed-field gel electrophoresis; WGS, whole-genome sequencing.

**Figure 4:**
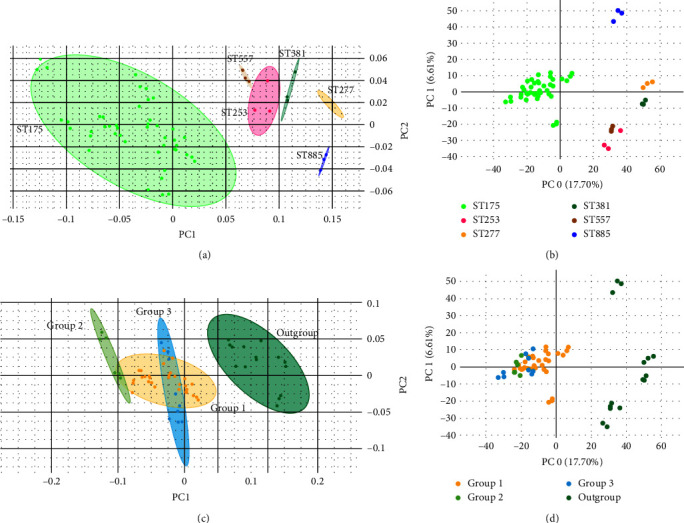
Principal component analysis scatter plot obtained by Fourier-transform infrared spectroscopy. (a) Plot according to sequence type (ST) of isolates obtained by IR Biotyper Software. (b) Plot according to ST obtained by Clover MS Data Analysis Software. (c) Plot according to whole-genome sequencing (WGS) outbreak groups obtained by IR Biotyper Software. (d) Plot according to WGS groups obtained by Clover MS Data Analysis Software.

**Figure 5:**
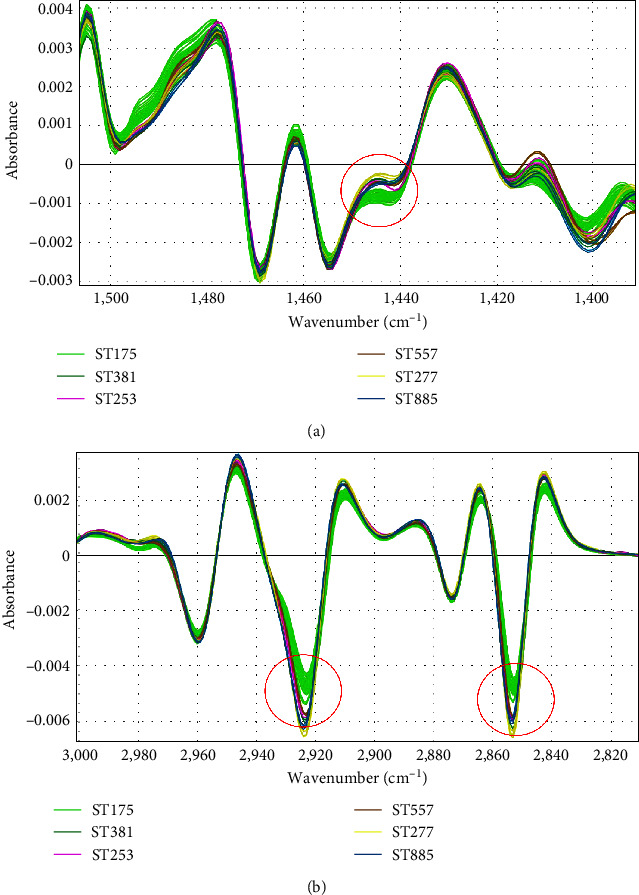
Fourier-transform infrared spectroscopy spectra of *Pseudomonas aeruginosa* isolates in the lipids region after applying second derivative. (a) 1,400–1,500 cm^−1^ region with different absorbance among outbreak and not-outbreak isolates (1,445 cm^−1^). (b) 2,800–3,000 cm^−1^ region with different absorbance among outbreak and not-outbreak isolates (2,925 and 2,955 cm^−1^).

After analyzing different spectral ranges, infrared absorbance in the lipid region (1,400–1,500 and 2,800–3,000 cm^−1^) showed the highest discriminatory power for the correct classification of *P. aeruginosa* outbreak strains. HCA using Euclidean distance and Ward linkage differentiated the outbreak strains (ST175) from control strains, which belonged to different sequence types (STs) such as ST227, ST253, ST381, ST557, and ST885 (Figure 3), with a discriminatory power similar to PFGE classification. In this case, the cut-off dendrogram distance for outbreak and controls differentiation was 0.350. For isolate differentiation, the automated cut-off distance assigned by IR Biotyper was 0.062. The two strains belonging to the same patient were clustered together with lower distance between them. Using PCA analysis in both IR Biotyper Software and Clover MS Data Analysis Software, ST175 strains were clustered together, distinctly from other STs (Figures 4(a) and 4(b)). The specific differences in infrared spectra absorbances among ST175 and other STs were found in 1,445 cm^−1^ (Figure 5(a)), 2,925 and 2,955 cm^−1^ (Figure 5(b)). The three subgroups inside the outbreak observed by WGS were not discriminated by HCA (Figure 3). In the PCA scatter plot (Figures 4(c) and 4(d)), although WGS Group 2 was partially separated from other groups, Groups 1 and 3 overlapped. Using PLS-DA and RF algorithm (Figure S4), it was also shown that Group 2 formed a distinct cluster, and the *k*-fold cross-validation obtained for differentiation of the three groups were 91.1% and 97.8%, respectively (Table S5).

## 4. Discussion

The implementation of MALDI-TOF MS and FTIR-S technology, combined with machine learning algorithms, allowed the correct classification of the MDR *P. aeruginosa* isolates causing a nosocomial outbreak in 2013–2014: 97.0% and 100% of the isolates were correctly classified by the algorithms applied to the protein peaks by MALDI-TOF MS with intensities above the established threshold (0.1) or to the specific biomarker peaks found with this technology, respectively. These results showed that MALDI-TOF MS yields a discrimination power similar to PFGE, the reference method for bacterial typing. Besides, the same protein spectra were further classified according to the information provided by WGS analysis. In this case, PLS-DA and SVM algorithms allowed a good classification of the *P. aeruginosa* isolates specifically correlated with the outbreak (Group 1) and showed that Group 2 was closely related with the outbreak strains, as the genomic analysis pointed out, and Group 3 and the control group were clearly unrelated to the outbreak. The validation of the classification models, carried out with 32 *P. aeruginosa* isolates characterized by ASO-PCR, yielded 90.0% correct classification of their protein spectra. Only three isolates were misclassified using the developed models, which indicates that the methodology described in this study may be applied as a rapid screening method when an outbreak is suspected. The implementation of FTIR-S technology showed the same discrimination power as PFGE to differentiate *P. aeruginosa* outbreak isolates. However, when the same level of classification provided by WGS technology was attempted with FTIR-S, the control group and outbreak Group 2 were clearly defined but Groups 1 and 3 overlapped. However, FTIR-S provided complementary information to the classification obtained with MALDI-TOF MS spectra by showing the correct classification of the different STs analyzed.

Previous studies have demonstrated that specific biomarker peaks present in the protein spectra obtained by MALDI-TOF MS could be used for the monitoring of *P. aeruginosa* STs. Cabrolier et al. [14] described a specific peak at 7,359 *m/z* that, combined with the absence of peaks at 7,329 and 12,154 *m/z* was specific of *P. aeruginosa* ST175. These results were further confirmed by Mulet et al. [29], who also found the peak at 7,359 *m/z* as a biomarker for *P. aeruginosa* ST175 and described another peak at 6,911 *m/z* as specific for this ST. In the present study, a peak at 6,915 *m/z* has been described as a biomarker of the *P. aeruginosa* outbreak strains belonging to the same ST. Although the difference of only 4 *m/z* between both peaks fall within the accepted margin of error of the applied pipeline (±4.5 *m/z*) and no other marker peak has been found in the area, further analysis is needed to confirm that both studies refer the same peak. Finally, the other two potential biomarker peaks described for the *P. aeruginosa* outbreak strains (5,169 and 7,236 *m/z*) have never been described before and, therefore, they might be outbreak-specific markers.

The reproducibility of this MALDI-TOF MS-based method for the rapid detection of a *P. aeruginosa* outbreak was evaluated. With the exception of the peak at 5,740 *m/z*, whose interday CV was just below 30.0%, the peaks located between 2,000 and 7,000 *m/z*—where the most common bacterial proteins locate—showed CV values ranging between 10.0% and 15.0% and have been considered as acceptable in previous studies [28]. The specific biomarker peaks that allowed the differentiation of the outbreak isolates showed an average intraday CV of 13.6% (range between 7.0% and 20.5%). Again, these values can be considered as acceptable. Only the biomarker peak at 6,915 *m/z* showed an interday CV variability CV of 32.5% (Figure S4(d)). Although this value is above the established limit (20.0%), the peak was always detected in *P. aeruginosa* outbreak strains regardless its intensity. Therefore, the presence of the 6,915 *m/z* peak can be reliably correlated with the outbreak along with the detection of the other two biomarker peaks at 5,169 and 7,236 *m/z*.

In recent years, FTIR-S has emerged as a reliable technology for outbreak analysis in clinical microbiology laboratories [30]. The simplicity and low costs of the sample preparation procedure and interpretation of results allows the follow-up of nosocomial outbreaks in real time since the turnaround time for the analysis of 30 isolates with this technology is approximately 3 hr. Although the most studied microorganism so far is *Klebsiella pneumoniae* [31], FTIR-S has been evaluated for typing other bacterial species such as *Salmonella* [19] or *Streptococcus pneumoniae* [20].

In our study, FTIR-S was able to discriminate *P. aeruginosa* outbreak isolates from nonoutbreak isolates at the same level than PFGE, either by HCA with a cut-off score of 0.350 or PCA (Figures 3 and 4). In addition, the two strains isolated from the same patient (numbers 139 and 143) were clustered together with very low distance, which indicates that this method recognizes them as the same strain and is reproducible (Figure 3). For ST differentiation, the lipid region (1,400–1,500 and 2,800–3,000 cm^−1^) showed the highest discriminatory power and allowed the correct classification of *P. aeruginosa* outbreak strains according to PFGE results. However, when this classification was compared with WGS information, the three outbreak subgroups were not clearly differentiated by FTIR-S by applying HCA. Similarly, the implementation of the PCA algorithm did not cluster the three groups of isolates separately, although Group 2 isolates were almost grouped apart (Figures 4(c) and 4(d)). When using PLS-DA and RF analyses for differentiation of these groups, it was also observed that Group 2 was clearly separated from the other outbreak groups according to WGS and the biggest differences among them were found in the 800–1,600 cm^−1^ region (Figure S5). It is important to note that WGS groups are based on SNP distance, and maybe these differences are not expressed phenotypically, and thus, FTIR-S clusters may not reflect differences detected by WGS. At the moment of writing, only one previous study has evaluated *P. aeruginosa* typing by FTIR-S in comparison to PFGE and MLST results [32]. The authors showed the reliability of the method for differentiation of STs with an optimal cut-off distance between 0.184 and 0.374. Besides, these results can be obtained in a turnaround time of 3 hr, a great advantage over PFGE.

One of the limitations of this study is the small number of strains available, but it is important to acknowledge that bacterial outbreaks usually involve a limited number of patients if they are well contained, thus making a larger number of samples unavailable for research purposes. The other limitation would be the limited number of isolates characterized by WGS. But, due to the costs of this technique, a more affordable approach (ASO-PCR) was carried out to classify the rest of the isolates.

However, despite these drawbacks, the results of this study showed that rapid diagnostic methods such as MALDI-TOF MS and FTIR-S may represent fast alternatives to conventional strategies—based on DNA sequencing—for real-time monitoring of nosocomial outbreaks, providing complementary information for the prompt characterization of the suspected isolates in a cost-efficient way. Although confirmation of the outbreak strains may request further analysis by WGS, the implementation and further validation of these rapid typing methods could help to reduce the number of isolates that require confirmation by expensive tests available to a limited number of microbiology laboratories.

## Data Availability

*Pseudomonas aeruginosa* sequences were deposited in the Pseudomonas Genome Database (http://www.pseudomonas.com). Proteomic data sourcing from MALDI-TOF and FTIR are available upon request.

## Supplementary Materials

Supplementary Materials Table S1: Description of the *P. aeruginosa* isolates included in this study and the techniques performed on them. Figure S1: Workflow of the *P. aeruginosa* isolates analysis with MALDI-TOF MS and FTIR S and the subsequent analysis of the spectra obtained by both methods. Table S2: Range of values explored for the automatic optimization of the balanced accuracy of model predictions obtained by the different methods of supervised training used. Table S3: Leave-one-out method for differentiation of outbreak and not-outbreak MALDI-TOF MS spectra by using the threshold matrix for PLS-DA, RF, NCA-KNN, and SVM algorithms. Table S4: Leave-one-out method for differentiation of WGS-groups in MALDI-TOF MS spectra by PLS-DA, SVM, and RF algorithms. Figure S2: Differentiation of outbreak strains according to whole-genome sequencing groups, using Clover MS Data Analysis Software and MALDI-TOF MS spectra. Figure S3: Reproducibility assay. Figure S4: Differentiation of outbreak strains according to whole-genome sequencing groups, using Clover MS Data Analysis Software and FTIR-S spectra. Table S5: Leave-one-out method for differentiation of whole-genome sequencing groups using PLS-DA and RF algorithms for FTIR-S spectra. Figure S5: Important regions in the FTIR spectra for strain discrimination.
