# Supplementary material for: Rapid Discrimination of Pseudomonas aeruginosa ST175 Isolates Involved in a Nosocomial Outbreak Using MALDI-TOF Mass Spectrometry and FTIR Spectroscopy Coupled with Machine Learning
Source: Transbound Emerg Dis. 2023 Sep 7;2023:8649429. doi: 10.1155/2023/8649429 (PMC12016727; doi:10.1155/2023/8649429)
Supplement: Supplementary Materials — Table S1: Description of the P. aeruginosa isolates included in this study and the techniques performed on them. Figure S1: Workflow of the P. aeruginosa isolates analysis with MALDI-TOF MS and FTIR S and the subsequent analysis of the spectra obtained by both methods. Table S2: Range of values explored for the automatic optimization of the balanced accuracy of model predictions obtained by the different methods of supervised training used. Table S3: Leave-one-out method for differentiation of outbreak and not-outbreak MALDI-TOF MS spectra by using the threshold matrix for PLS-DA, RF, NCA-KNN, and SVM algorithms. Table S4: Leave-one-out method for differentiation of WGS-groups in MALDI-TOF MS spectra by PLS-DA, SVM, and RF algorithms. Figure S2: Differentiation of outbreak strains according to whole-genome sequencing groups, using Clover MS Data Analysis Software and MALDI-TOF MS spectra. Figure S3: Reproducibility assay. Figure S4: Differentiation of outbreak strains according to whole-genome sequencing groups, using Clover MS Data Analysis Software and FTIR-S spectra. Table S5: Leave-one-out method for differentiation of whole-genome sequencing groups using PLS-DA and RF algorithms for FTIR-S spectra. Figure S5: Important regions in the FTIR spectra for strain discrimination. [file 8649429.f1.docx]

**Table S1.** Description of the *P. aeruginosa* isolates included in this study and the techniques performed on them.

| **AST** | **STRAIN ID** | **MALDI-TOF** | **FTIR-S** | **PFGE** | **WGS** | **ASO-PCR** | **AST** |
| --- | --- | --- | --- | --- | --- | --- | --- |
|  |  | **n=67** | **n=20** | **n=20** | **n=35** | **n=32** | **n=67** |
| **CONTROLS** | 132 | Yes | Yes | Yes (Control) | Yes (Control) | No | non-MDR |
| **Not-outbreak** | 7488 | Yes | Yes | Yes (Control) | Yes (Control) | No | MDR |
|  | 130 | Yes | Yes | Yes (Control) | Yes (Control) | No | non-MDR |
|  | 131 | Yes | Yes | Yes (Control) | Yes (Control) | No | non-MDR |
|  | 152 | Yes | Yes | Yes (Control) | Yes (Control) | No | non-MDR |
|  | 295 | Yes | No | No | Yes (Control) | No | MDR |
|  | 290 | Yes | No | No | Yes (Control) | No | MDR |
|  | 287 | Yes | No | No | Yes (Control) | No | MDR |
|  |  |  |  |  |  |  |  |
| **GROUP 1** | 151 | Yes | Yes | Yes (Outbreak) | Yes (Outbreak) | No | MDR |
|  | 2184 | Yes | Yes | Yes (Outbreak) | Yes (Outbreak) | No | MDR |
|  | 145 | Yes | Yes | Yes (Outbreak) | Yes (Outbreak) | No | MDR |
|  | 139 | Yes | Yes | Yes (Outbreak) | Yes (Outbreak) | No | MDR |
|  | 143 | Yes | Yes | Yes (Outbreak) | Yes (Outbreak) | No | MDR |
|  | 144 | Yes | Yes | Yes (Outbreak) | Yes (Outbreak) | No | MDR |
|  | 138 | Yes | No | No | Yes (Outbreak) | No | MDR |
|  | 146 | Yes | Yes | Yes (Outbreak) | Yes (Outbreak) | No | MDR |
|  | 142 | Yes | Yes | Yes (Outbreak) | Yes (Outbreak) | No | MDR |
|  | 141 | Yes | Yes | Yes (Outbreak) | Yes (Outbreak) | No | MDR |
|  | 1031 | Yes | Yes | Yes (Outbreak) | Yes (Outbreak) | No | MDR |
|  | 1680 | Yes | No | No | Yes (Outbreak) | No | MDR |
|  | 137 | Yes | No | No | Yes (Outbreak) | No | MDR |
|  | 3478 | Yes | No | No | Yes (Outbreak) | No | MDR |
|  | 4264 | Yes | No | No | Yes (Outbreak) | No | MDR |
|  | 1971 | Yes | No | No | Yes (Outbreak) | No | MDR |
|  | 5321 | Yes | No | No | Yes (Outbreak) | No | MDR |
|  | 296 | Yes | No | No | Yes (Outbreak) | No | MDR |
|  | 288 | Yes | No | No | Yes (Outbreak) | No | MDR |
|  | 291 | Yes | No | No | Yes (Outbreak) | No | MDR |
|  | 292 | Yes | No | No | Yes (Outbreak) | No | MDR |
|  |  |  |  |  |  |  |  |
| **GROUP2** | 148 | Yes | Yes | Yes (Outbreak) | Yes (Control) | No | MDR |
|  | 147 | Yes | Yes | Yes (Outbreak) | Yes (Control) | No | MDR |
|  |  |  |  |  |  |  |  |
| **GROUP 3** | 149 | Yes | Yes | Yes (Outbreak) | Yes (Control) | No | MDR |
|  | 129 | Yes | Yes | Yes (Outbreak) | Yes (Control) | No | MDR |
|  | 150 | Yes | Yes | Yes (Outbreak) | Yes (Control) | No | MDR |
|  | 169539 | Yes | No | No | Yes (Control) | No | MDR |
|  |  |  |  |  |  |  |  |
| **ASO-PCR** | 790-12 | Yes | No | No | No | Yes (Outbreak) | non-MDR |
| **Not-outbreak** | 565-13 | Yes | No | No | No | Yes (Outbreak) | non-MDR |
|  | 882-13 | Yes | No | No | No | Yes (Outbreak) | non-MDR |
|  | 4138-15 | Yes | No | No | No | Yes (Outbreak) | non-MDR |
|  | 4292-15 | Yes | No | No | No | Yes (Outbreak) | MDR |
|  | 919-16 | Yes | No | No | No | Yes (Outbreak) | non-MDR |
|  | 117-16 | Yes | No | No | No | Yes (Outbreak) | non-MDR |
|  | 1,7E+07 | Yes | No | No | No | Yes (Outbreak) | MDR |
|  | 1054-17 | Yes | No | No | No | Yes (Outbreak) | non-MDR |
|  | 1070-17 | Yes | No | No | No | Yes (Outbreak) | non-MDR |
|  | 1455-17 | Yes | No | No | No | Yes (Outbreak) | non-MDR |
|  | 1505-17 | Yes | No | No | No | Yes (Outbreak) | non-MDR |
|  | 57-17 | Yes | No | No | No | Yes (Outbreak) | non-MDR |
|  | 772-17 | Yes | No | No | No | Yes (Outbreak) | MDR |
|  | 921-17 | Yes | No | No | No | Yes (Outbreak) | MDR |
|  | 1503-17 | Yes | No | No | No | Yes (Outbreak) | non-MDR |
|  |  |  |  |  |  |  |  |
| **ASO-PCR** | 1,2E+07 | Yes | No | No | No | Yes (Control) | MDR |
| **Outbreak** | 1983-13 | Yes | No | No | No | Yes (Control) | MDR |
|  | 2645-15 | Yes | No | No | No | Yes (Control) | MDR |
|  | 836-16 | Yes | No | No | No | Yes (Control) | MDR |
|  | 1626-17 | Yes | No | No | No | Yes (Control) | MDR |
|  | 1,7E+07 | Yes | No | No | No | Yes (Control) | MDR |
|  | 1093-17 | Yes | No | No | No | Yes (Control) | MDR |
|  | 1453-17 | Yes | No | No | No | Yes (Control) | MDR |
|  | 1512-17 | Yes | No | No | No | Yes (Control) | MDR |
|  | 1565-17 | Yes | No | No | No | Yes (Control) | MDR |
|  | 1677-17 | Yes | No | No | No | Yes (Control) | MDR |
|  | 1862-17 | Yes | No | No | No | Yes (Control) | MDR |
|  | 1928-17 | Yes | No | No | No | Yes (Control) | MDR |
|  | 1964-17 | Yes | No | No | No | Yes (Control) | MDR |
|  | 229-17 | Yes | No | No | No | Yes (Control) | MDR |
|  | 378-17 | Yes | No | No | No | Yes (Control) | MDR |

**
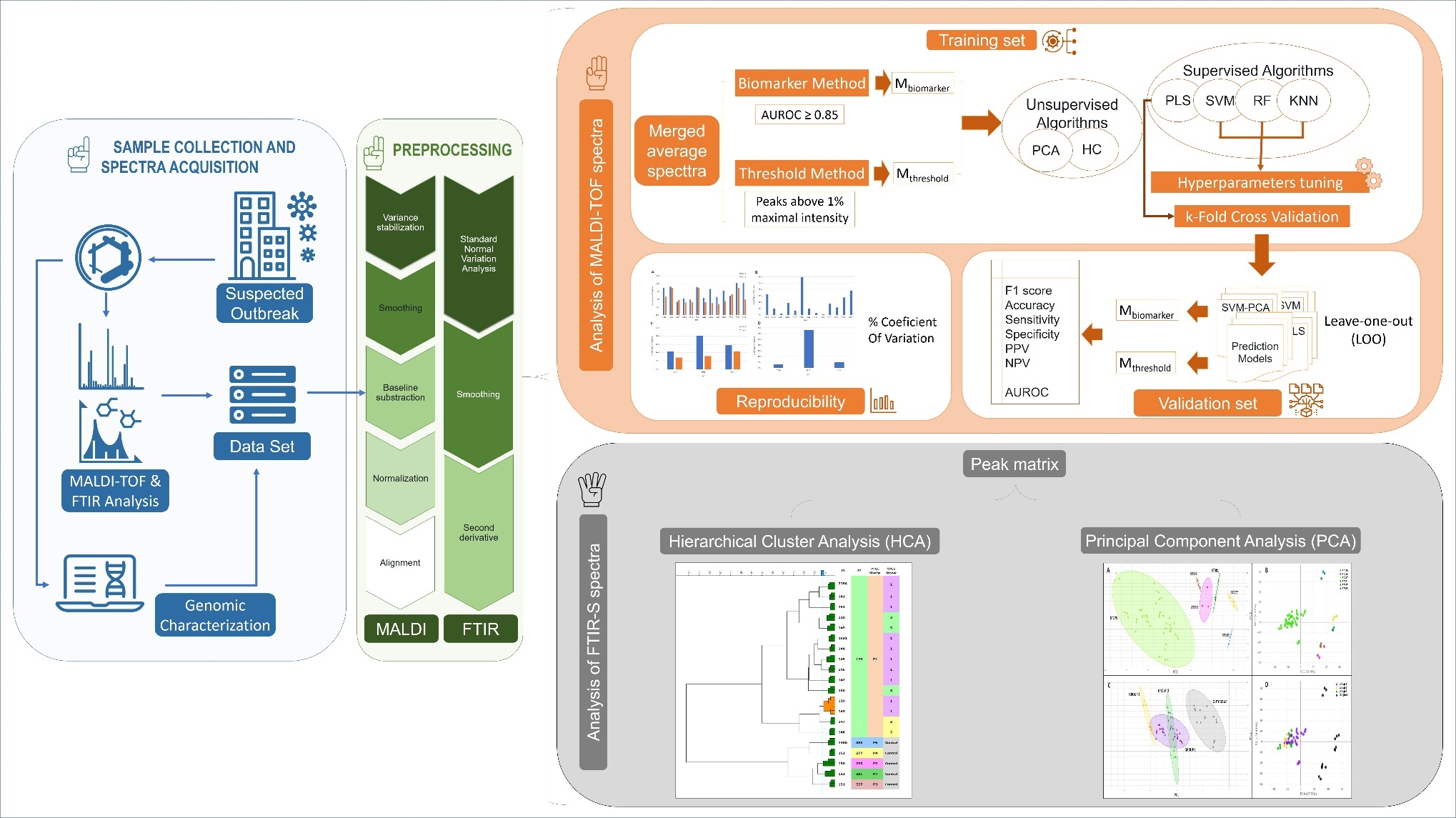
**

**Figure S1.** Workflow of the *P. aeruginosa* isolates analysis with MALDI-TOF MS and FTIR S and the subsequent analysis of the spectra obtained by both methods

**Table S2**. Range of values explored for the automatic optimization of the balanced accuracy of model predictions obtained by the different methods of supervised training used.

| Supervised method | Hyperparameter | Range of values |
| --- | --- | --- |
| SVM | C | {1e-5, 1e-4, 1e-3, 1e-2, 1e-1, 1, 10} |
| NCA-KNN | K | Odd values in [3, 21] |
| RF | Number of estimators | {50, 100, 200, 400} |
|  | Number of features to look for the best split | Square root or logarithm in base 2 of the total number of features. |
|  | Maximum depth of each tree | {10, 30, 50, 70, 110} |
|  | Minimum number of samples required to split an internal node | {2, 5, 10} |
|  | Minimum number of samples required to be a leaf node | {1, 2, 4} |

**Table S3.** Leave-one-out method for differentiation of outbreak and not-outbreak MALDI-TOF MS spectra by using the threshold matrix for PLS-DA, RF, NCA-KNN and SVM algorithms. RF hyperparameters: 50 Estimators, 20 Max Features, 10 Max Depth, 2 Min split size and 1 Min Samples per leaf. PLS-DA components: 3. SVM hyperparameter C: 0.00001 (liblinear kernel). Hyperparameter C is the trade-off value of misclassified samples. PCA-SVM hyperparameter: 10 (liblinear kernel). NCA-KNN number of neighbors: 3

| **Actual\Predicted** | Outbreak | Not outbreak | **% Correct** |
| --- | --- | --- | --- |
| **PLS-DA, PCA-SVM, RF and NCA-KNN** |  |  |  |
| Outbreak | 27 | 0 | 100% |
| Not Outbreak | 1 | 7 | 87.5% |
| **PLS-DA, PCA-SVM, RF and NCA-KNN Accuracy / Balanced Accuracy** | | | 97.1%/93.75% |
| **SVM** |  |  |  |
| Outbreak | 26 | 1 | 96.3% |
| Not Outbreak | 1 | 7 | 87.5% |
| **SVM Accuracy / Balanced Accuracy** | | | 94.3%/91.9% |

**Table S4.** Leave-one-out method for differentiation of WGS-groups in MALDI-TOF MS spectra by PLS-DA, SVM and RF algorithms. RF hyperparameters: 50 Estimators, 20 Max Features, 10 Max Depth, 2 Min split size and 1 Min Samples per leaf. PLS-DA components: 3. SVM hyperparameter C: 0.01 (liblinear kernel). Hyperparameter C is the trade-off value of misclassified samples. NCA-KNN number of neighbors: 3

| **Actual\Predicted** | Group 1 | Group 2 | Group 3 | Not Outbreak | **% Correct** |
| --- | --- | --- | --- | --- | --- |
| **PLS-DA** |  |  |  |  |  |
| Group 1 | 21 | 0 | 0 | 0 | 100% |
| Group 2 | 2 | 0 | 0 | 0 | 0% |
| Group 3 | 1 | 0 | 3 | 0 | 75% |
| Not Outbreak | 2 | 0 | 0 | 6 | 75% |
| **PLS-DA Accuracy / Balanced Accuracy** | | |  |  | 85.7%/62.5% |
| **RF** |  |  |  |  |  |
| Group 1 | 21 | 0 | 0 | 0 | 100% |
| Group 2 | 2 | 0 | 0 | 0 | 0% |
| Group 3 | 1 | 0 | 3 | 0 | 75% |
| Not Outbreak | 1 | 0 | 0 | 7 | 87.5% |
| **RF Accuracy / Balanced Accuracy** | | |  |  | 88.57%/65.63% |
| **SVM** |  |  |  |  |  |
| Group 1 | 21 | 0 | 0 | 0 | 100% |
| Group 2 | 1 | 1 | 0 | 0 | 50% |
| Group 3 | 0 | 0 | 4 | 0 | 100% |
| Not Outbreak | 0 | 0 | 1 | 7 | 87.5% |
| **SVM Accuracy / Balanced Accuracy** | | |  |  | 94.3%/84.38% |
| **NCA- KNN** |  |  |  |  |  |
| Group 1 | 21 | 0 | 0 | 0 | 100% |
| Group 2 | 2 | 0 | 0 | 0 | 0% |
| Group 3 | 1 | 0 | 3 | 0 | 75% |
| Not Outbreak | 1 | 0 | 1 | 6 | 75% |
| **NCA-KNN Accuracy / Balanced Accuracy** | | |  |  | 85.7%/62.5% |


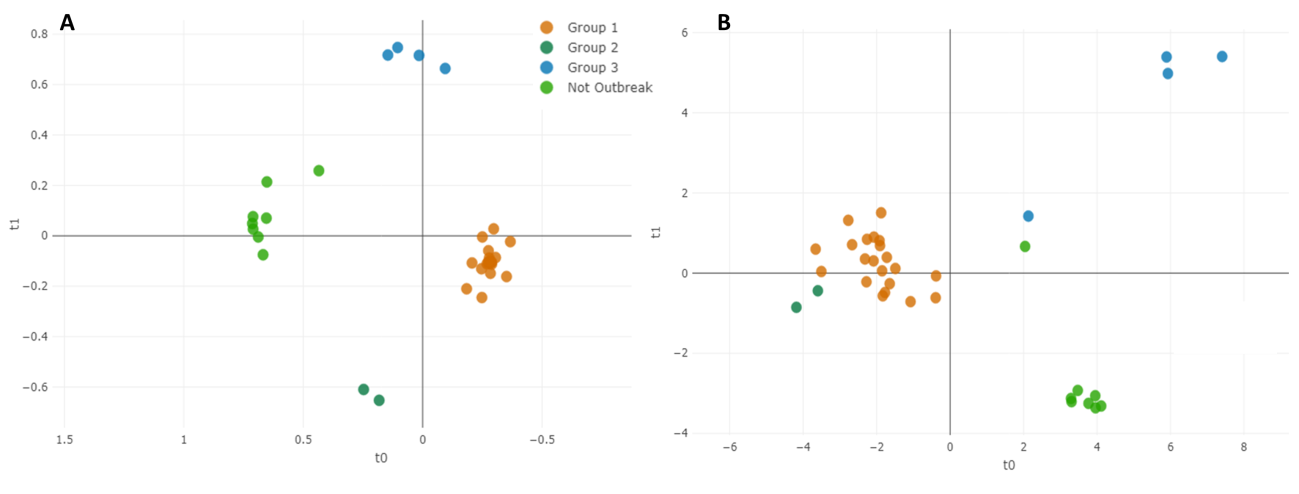
**Figure S2.** Differentiation of outbreak strains according to Whole-genome sequencing groups, using Clover MS Data Analysis software and MALDI-TOF MS spectra. **A:** Random Forest scatter plot. **B:** Neighborhood Component Analysis-K Nearest Neighbors scatter plot.


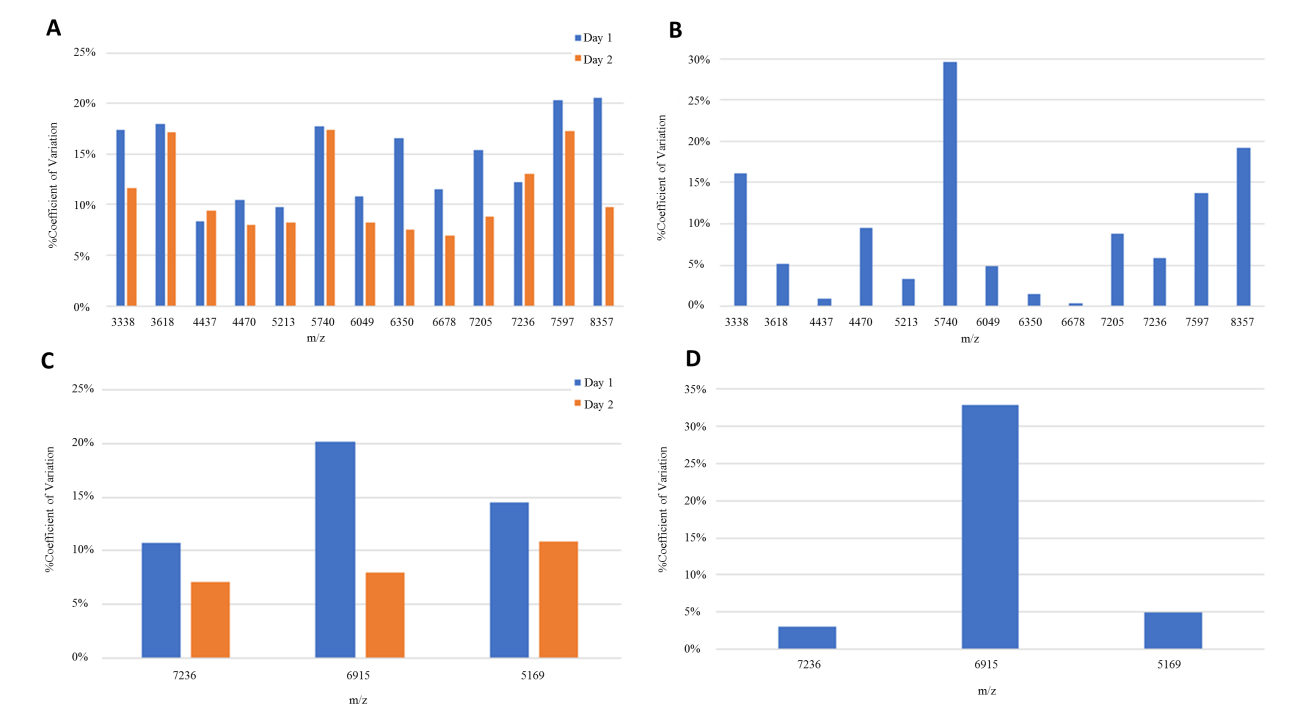


**Figure S3.** Reproducibility assay. **A:** Coefficient of Variation (CV) of most common peaks each day (intra-day reproducibility); **B:** CV of most common peaks between both days (inter-day reproducibility); **C:** CV of biomarker peaks each day (intra-day reproducibility); **D:** CV of biomarker peaks between both days (inter-day reproducibility).


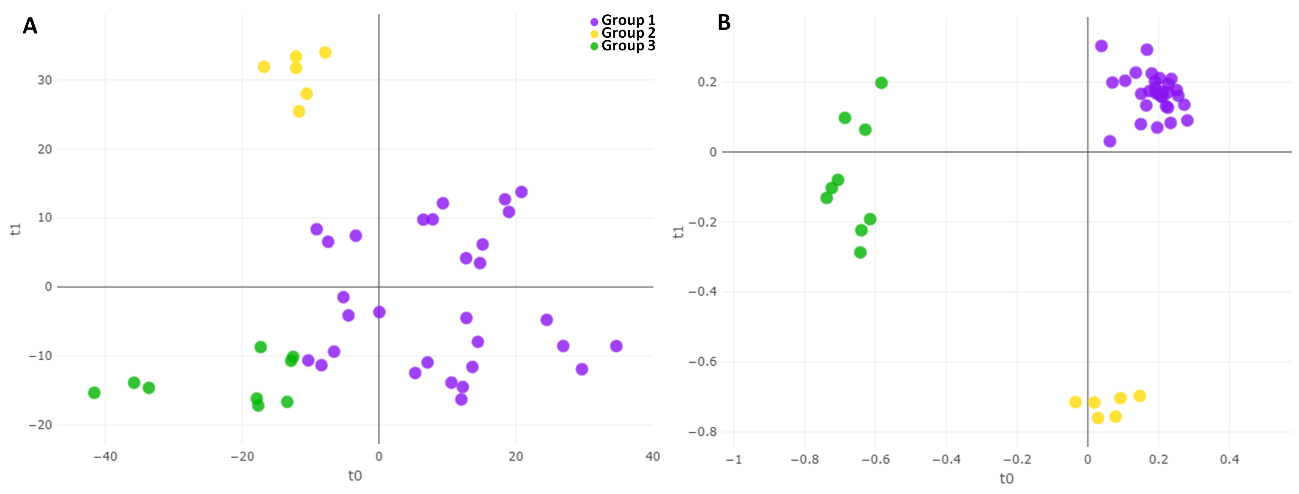


**Figure S4.** Differentiation of outbreak strains according to Whole-genome sequencing groups, using Clover MS Data Analysis software and FTIR-S spectra. **A:** Partial Least Squares-Discriminant Analysis scatter plot. **B:** Random Forest scatter plot.

**Table S5** Leave-one-out method for differentiation of Whole-genome sequencing groups using PLS-DA and RF algorithms for FTIR-S spectra. PLS-DA Components: 3, RF hyperparameters: 50 estimators, 59 max. features, 10 max. depth, 5 min. split size and 1 min. samples per leaf.

| **Actual\Predicted** | Group 1 | Group 2 | Group 3 | **% Correct** |
| --- | --- | --- | --- | --- |
| **PLS-DA** |  |  |  |  |
| Group 1 | 30 | 0 | 0 | 100% |
| Group 2 | 1 | 5 | 0 | 83.3% |
| Group 3 | 4 | 0 | 5 | 55.56% |
| **PLS-DA Accuracy / Balanced Accuracy** | |  |  | 91.11%/79.63% |
| **RF** |  |  |  |  |
| Group 1 | 30 | 0 | 0 | 100% |
| Group 2 | 0 | 6 | 0 | 100% |
| Group 3 | 1 | 0 | 8 | 88.89% |
| **RF Accuracy / Balanced Accuracy** | |  |  | 97.78% / 96.29% |


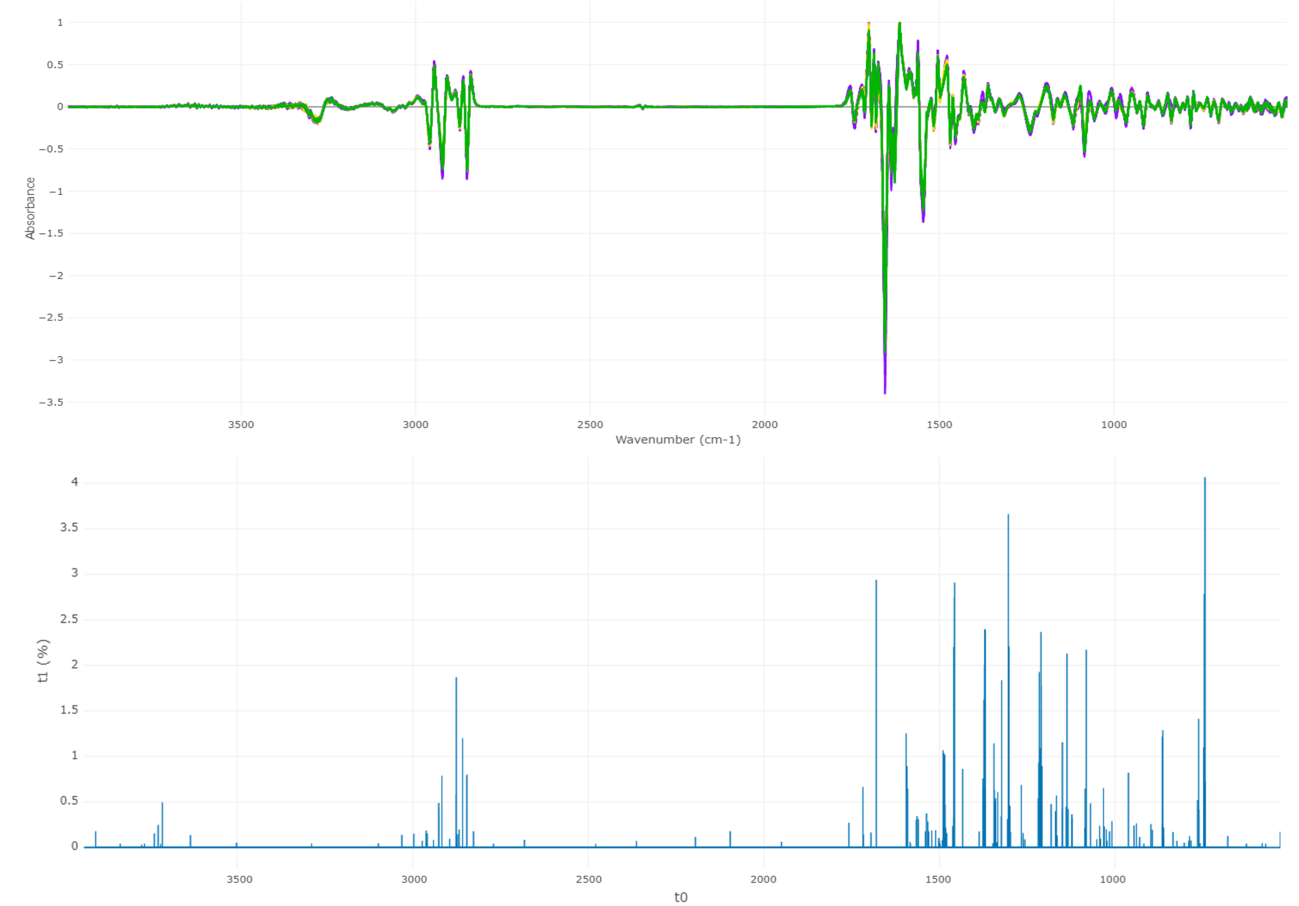


**Figure S5.** Important regions in the FTIR spectra for strain discrimination. **Up:** Second derivate of FTIR-S spectra according to Whole-genome sequencing groups (Group 1: purple; Group 2: yellow; Group 3: green). **Down:** Important peaks for the differentiation of P. aeruginosa isolates according to Whole-genome sequencing using Random Forest for FTIR-S spectra.
